# Physiochemical and Electrochemical Properties of a Heat-Treated Electrode for All-Iron Redox Flow Batteries

**DOI:** 10.3390/nano14090800

**Published:** 2024-05-05

**Authors:** Nitika Devi, Jay N. Mishra, Prabhakar Singh, Yong-Song Chen

**Affiliations:** 1 Department of Mechanical Engineering and Advanced Institute of Manufacturing with High-tech Innovations, National Chung Cheng University, 168, University Rd., Minhsiung Township, Chiayi County 621301, Taiwan; imeysc@ccu.edu.tw; 2 Department of Physics, Indian Institute of Technology, Varanasi 221005, India; jaynarayanmishra.rs.phy20@itbhu.ac.in (J.N.M.); psingh.app@iitbhu.ac.in (P.S.)

**Keywords:** iron redox flow battery, electrode heat treatment, specific capacitance, energy efficiency

## Abstract

Iron redox flow batteries (IRFBs) are cost-efficient RFBs that have the potential to develop low-cost grid energy storage. Electrode kinetics are pivotal in defining the cycle life and energy efficiency of the battery. In this study, graphite felt (GF) is heat-treated at 400, 500 and 600 °C, and its physicochemical and electrochemical properties are studied using XPS, FESEM, Raman and cyclic voltammetry. Surface morphology and structural changes suggest that GF heat-treated at 500 °C for 6 h exhibits acceptable thermal stability while accessing the benefits of heat treatment. Specific capacitance was calculated for assessing the wettability and electrochemical properties of pristine and treated electrodes. The 600 °C GF has the highest specific capacitance of 34.8 Fg^−1^ at 100 mV s^−1^, but the 500 °C GF showed the best battery performance. The good battery performance of the 500 °C GF is attributed to the presence of oxygen functionalities and the absence of thermal degradation during heat treatment. The battery consisting of 500 °C GF electrodes offered the highest voltage efficiency of ~74%, Coulombic efficiency of ~94%, and energy efficiency of ~70% at 20 mA cm^−2^. Energy efficiency increased by 7% in a battery consisting of heat-treated GF in comparison to pristine GF. The battery is capable of operating for 100 charge–discharge cycles with an average energy efficiency of ~ 67% for over 100 cycles.

## 1. Introduction

Energy storage is a critical area of research due to the continuous increase in energy demand and the depletion of fossil fuels [1]. Devices like supercapacitors, fuel cells, and batteries have been developed for generating and storing energy from various energy resources [2,3]. Redox flow batteries (RFBs) are a type of aqueous batteries that generate electricity through redox reactions of electrolytes. RFBs have many advantages over conventional batteries like the highest lifetime and cycle time, extremely low self-discharge, a depth of discharge that does not affect the cycle lifetime, etc. In vanadium redox flow batteries (VRFBs), the depth of discharge is approximately 100%, which is limited to only 50-80% in lead acid batteries and lithium-ion batteries. Like any device, RFBs also have some limitations such as low energy density due to an aqueous system, which makes them non-suitable for mobile applications. Also, energy efficiency is low compared to lithium-ion batteries, but the difference can be considerably improved when working on large units. Therefore, RFBs are a suitable option for large-scale storage applications [4]. Application areas of RFBs are mainly related to peak load balancing and integration with wind and photovoltaic power sources for large-scale energy storage [5]. In addition, RFBs overcome drawbacks associated with conventional batteries, such as cost- and safety-related issues. Redox reactions between two electroactive molecules are used as a means of storing energy in RFBs. Basic components of the RFBs are two half-cells containing positive and negative electrolytes, electrodes, and an ion-exchange membrane. The membrane is used to separate the reactions in two half-cells and for transporting ions [6,7]. Scientists have developed Fe/Cr, Zn/Cl, Zn/Br, H/Br, V/V and S/Br redox-coupled RFBs. However, only Zn/Br [8] and VRFBs [9] have showed potential for meeting commercial energy demand. Among these, VRFBs have gained considerable attention due to fewer irreversible losses resulting from the variable oxidation states of vanadium. Moreover, VRFBs are free from toxic fumes and membrane fouling problems, providing high energy efficiency [10]. However, some challenges need to be addressed to fully leverage the advantages of these batteries, such as high cost and the oxidation problem of V^2+^ ions. As a result, VRFBs may not be a suitable option for commercialization due to their high cost [11]. Since abundant iron can alleviate the cost issue, all-iron redox flow batteries (IRFBs) can be considered a viable substitute for VRFBs. Fe^3+^/Fe^2+^ and Fe^2+^/Fe^0^ are two redox pairs in IRFBs that store energy using subsequent redox reactions [12].

Negative: $Fe^{0}\overset{Disch\arg e}{\underset{Ch\arg e}{⇄}}Fe^{2+}+2e^{-}$ *E^0^* = −0.44 V vs RHE

Positive: 2$Fe^{3+}+2e^{-}\overset{\mathrm{Discharge}}{\underset{\mathrm{Charge}}{⇄}}2Fe^{2+}$ *E^0^* = +0.77 V vs RHE

Overall:  $Fe^{0}+2Fe^{3+}\overset{\mathrm{Discharge}}{\underset{\mathrm{Charge}}{⇄}}3Fe^{2+}$ *E^0^* = −1.21 V vs RHE

IRFBs have advantages over VRFBs, and their high utility, low chemical toxicity, and lower cost make them a suitable choice for commercialization. Although IRFBs have the merit of cost-effectiveness, there are limitations like low energy density, iron plating, and hydrogen evolution reaction (HER). For a good all-iron redox flow battery (IRFB) performance, suppressing HER is important as Fe^2+^/Fe^0^ occurs at a negative potential (–0.44 V), which is more negative than HER at pH = 0 [13]. Other than this, iron deposition is another challenge in IRFB, as metallic iron formed during battery reactions is not easily dissolved into the electrolyte due to iron hydroxide precipitation. These side reactions result in the low energy density of IRFBs [14]. Each component of battery assembly has a considerable effect on the battery perfromance [15,16]. 

Electrode properties can significantly affect the RFB performance, as redox reactions occur on the electrode surface, so an electrode with good electrocatalytic properties has more potential for RFBs. Carbon in different forms such as carbon fibre, carbon felt, and carbon cloth has been utilized as an electrode for RFBs. Usually, these electrodes have very low electrochemical reaction kinetics, which results in sluggish redox reactions and degrades the battery performance [17]. Scientists have been making different efforts to improve the electrocatalytic properties of carbon-based electrodes, including thermal treatment [18], chemical treatment [19], heteroatom doping [20] and decoration with various nanoparticles [21]. Thermal and acid treatments result in adding the number of oxygen functional groups on the electrode surface, which has a positive effect on the electrochemical properties of the electrode. A good catalytic properties electrode can help in suppressing the HER reaction and precipitation of electrolyte due to iron hydroxide formation. Pezeshki et al. [22] reported that the electrocatalytic properties of the electrode significantly improved due to oxygen-enriched thermal activation. Improved electrocatalytic electrodes increased the round-trip energy efficiency (EE) of VRFB by 10%. Heat treatment methods and conditions also influence the electrode properties. Eifert et al. [23] studied the effect of treatment methods like thermal treatment, chemical ageing, and electrochemical ageing on the electrode properties. The study extensively studied the surface chemistry of the treated electrode and observed that the treatment conditions and type of carbon electrode can alter the electrode surface chemistry significantly. Wu et al. [24] applied microwave heat treatment on graphite felt (GF), and modified electrodes were utilized in VRFB. Microwave heat treatment inserted hydrophilic functional groups on its surface defects, which was responsible for the enhanced electrochemical behavior of the GF electrodes. There are many studies that have demonstrated the electrode surface modification effect on the VRFBs, but very few discuss the performance effect on IRFBs. Lim et al. [25] reported the effect of heat treatment on the performance of a GF electrode with ferrocyanide and an iron-3-[Bis(2-hydroxyethyl)amino]−2-hydroxy-propanesulfonic acid complex (Fe(DIPSO)) as a redox couple for IRFB. This study discussed only the electrochemical aspect of heat treatment and did not comment on anything regarding the physiochemical aspects of the electrode and how it can affect various aspects of battery. Also, electrolytes employed in the mentioned study were different from those in the present study.

In this study, PAN-based GF electrodes were heat-treated at temperatures of 400, 500, and 600 °C for 6 h. Study examined the physiochemical and electrochemical alterations of various heat-treated GFs corresponding to various heating temperatures. Heat-treated GFs were further tested in all IRFBs at different current densities and for 100 charge–discharge cycles. This study will help in understanding the roles of the morphological and electrochemical properties of the electrode in defining IRFB performance. 

## 2. Materials and Methods

### 2.1. Heat Treatment of GFs

Heat treatment was applied to a commercially purchased PAN-based GF (pristine GF) (GF650, CeTech Co., Ltd., Taichung City, Taiwan) electrode at three different temperatures: 400 °C (400 °C GF), 500 °C (500 °C GF) and 600 °C (600 °C GF), with a heating rate of 10 degrees per minute for 6 h. The heating process was conducted in a muffle furnace. To investigate the effects of heat treatment time, the electrode treated at 500 °C was subjected to varied heating durations of 5, 6 and 7 h.

### 2.2. Electrolyte Preparation and Cell Formation

The separator of IRFB was an 50 µm non-reinforced anion-exchange membrane (fumasep® FAP-450- PET, Fumatech, Ludwigsburg, Germany). The compositions of the positive and negative electrolytes were 0.5 M FeCl_2_.4H_2_O (98%, Alfa Aesar, Ward Hill, MA, USA), 1.5 M NH_4_Cl (99.5%, PanReac AppliChem, Darmstadt, Germany), 0.5 M C_6_H_5_Na_3_O_7_ (99%, thermo scientific, Waltham, MA, USA), and 1 M FeCl_2_.4H_2_O and 1.5 M NH_4_Cl dissolved in distilled water, respectively. The volume used for positive and negative electrolytes was 50 mL in each experiment. The electrolyte flow rate was regulated using two peristaltic pumps. Electrolyte flow rate for all experiments was 1.5 L h^−1^. A battery tester (PFX2011S, Kikusui Electronics Corp., Yokohama, Japan) was used to evaluate the performance of IRFB. All experiments were conducted at the current density of 20 mA cm^−2^ with lower and cut-off voltages of 0.8 V and 1.5 V, respectively. A cell consisting of an anion-exchange membrane, heat-treated electrodes, graphite plates, gold-coated copper current collectors, and end plates, with an active area of 5 × 5 cm^2^, was used for performance measurement. A comparable cell with a pristine GF electrode was used for battery comparison.

### 2.3. Characterization of Electrodes

Thermogravimetric analysis (TGA) measurements were performed on a TGA-50 (M/s Shimadzu (Asia Pacific) Pte Ltd., Singapore, Singapore) using platinum pans under a 50 mL min^−1^ flow of air. GFs were tested from room temperature until 900 °C with a ramp of 10 °C min^−1^. FESEM images of pristine GF and heat-treated GF were obtained via a Hitachi FE-SEM, S-4800 (Hitachi FE-SEM, S-4800, Montreal, Quebec, Canada). The Raman spectra of the samples were examined with the LabRAM HR Evol Raman spectrometer (Lab RAM HR, Lille, France). Contact angle measurement was performed by using Cam 100 and Creating Nano Technologies Inc. (contact angle CAM 100−i). The nitrogen adsorption–desorption isotherm at 77 K, pore-size distribution, and Brunauer–Emmett–Teller (BET) surface area were obtained using BELLSORP MAX II and BELCAT-II (MicrotracBEL Corp. br, Osaka, Japan). Using a Mg Kα source (1253.6 eV) and X-ray photoelectron spectroscopy (XPS) measurements (CLAM4 electron analyzer from Thermo VG scientific), the elemental compositions and chemical states of the elements in the sample surfaces were ascertained. 

### 2.4. Electrochemical Test

Electrochemical measurements of various heat-treated electrodes were performed using a potentiostat (CHI700E, Austin, TX, USA) in an electrolyte containing 1 M FeCl_2_.4H_2_O and 1.5 M NH_4_Cl. Cyclic voltammetry (CV) was performed at scan rates of 40, 60, 80 and 100 mV s^−1^ in a wide voltage window from 0.4 to 1.6 V. The area of the pristine GF and heat-treated GF working electrode was 1 × 1 cm^2^. Electrochemical cell was completed using a Pt counter electrode and Ag/AgCl filled with 3 M KCl as a reference electrode.

## 3. Results and Discussion

### 3.1. Structural and Morphological Characterizations

**Figure 1:**
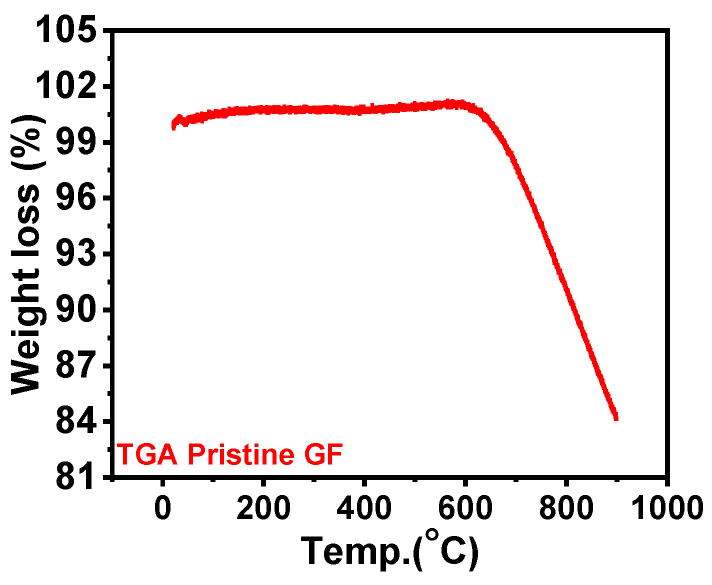
TGA analysis of pristine GF in air atmosphere.

**Figure 2:**
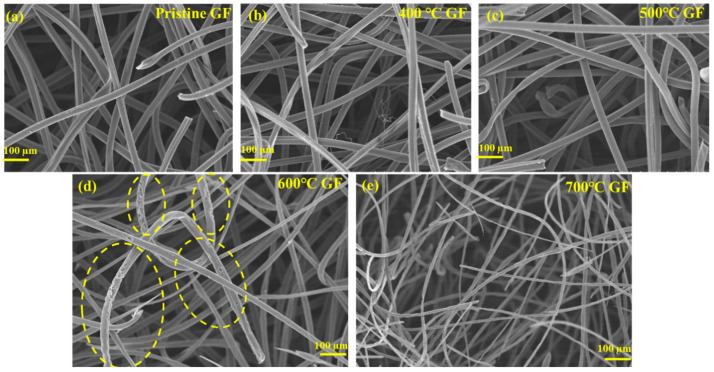
FESEM images of (**a**) pristine GF, (**b**) 400 °C GF, (**c**) 500 °C GF, (**d**) 600 °C GF and (**e**) 700 °C GF.

TGA of pristine GF was carried out to study its thermal stability and resulting temperature vs. weight loss variations, as shown in Figure 1. Figure 1 shows that GF degrading begins after 600 °C because of carbon oxidation. This observation aligned with published reports, which suggested that GF’s mechanical stability and structure are significantly compromised above 600 °C [26,27]. A slight mass loss occurred near 400 °C, attributed to the removal of moisture or impurities. After 400 °C, a slight weight gain occurred due to the insertion of oxygen functionalities, a phenomenon further discussed in detail using XPS and Raman spectroscopy. Structural changes were evident from the FESEM images of different heat-treated GFs at various temperatures, as shown in Figure 2. FESEM images suggested that for pristine GF (Figure 2a), 400 °C GF (Figure 2b), and 500 °C GF (Figure 2c), the morphology closely always resembles the pristine GF, indicating that heat treatment did not damage the material structure.

In Figure 2d, the highlighted spots in the FESEM image of 600 °C GF are indicating felt material degradation. A GF was heat-treated at 700 °C for 6 h (700 °C GF) and studied via FESEM to identify further details about thermal degradation. The FESEM image of 700 °C GF (Figure 2e) revealed that strands of GF appear significantly thinner compared to the pristine and other heat-treated graphite GFs. This suggests substantial weight loss at this temperature due to GF degradation, resulting from extensive oxidation of the carbon material. For further analysis, only GFs heat-treated up to 600 °C were considered, as beyond this temperature, a notable degradation was observed with significantly decreased mechanical strength. 

**Figure 3:**
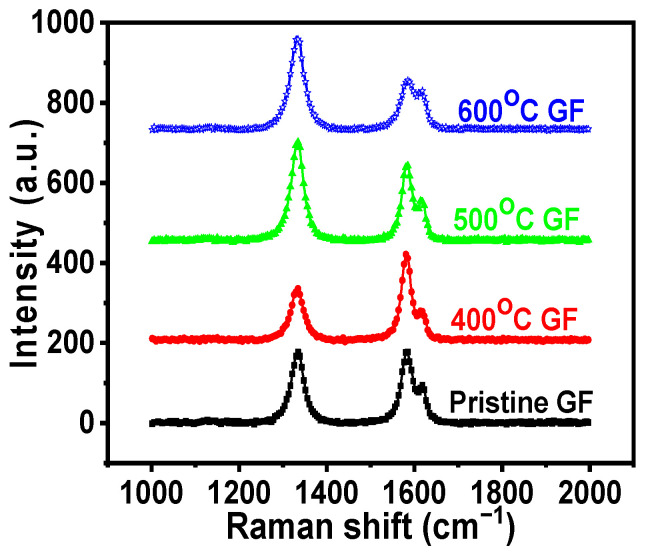
Raman spectra of pristine GF and various heat-treated GFs.

Surface defects resulting from heat treatment were further studied using Raman Spectroscopy. The Raman spectra of pristine GF and heat-treated GFs are shown in Figure 3. Raman spectra of various GFs exhibit two characteristic peaks, D and G, corresponding to defects in carbon materials and C-C vibrations of sp^2^ hybridization, respectively. In carbon materials, D and G bands exhibited Raman shifts of 1350 cm^−1^ and 1580 cm^−1^, respectively [28,29]. The heat treatment of GF results in defects, as indicated by the increased peak intensity of D and G bands with rising temperatures [30]. The I_D_/I_G_ ratio indicates the extent of defects present in material and was observed to increase with higher-temperature treatment. Table 1 displays the I_D_/I_G_ ratios for different heat-treated GFs. I_D_/I_G_ ratio of pristine GF was 0.89, which increased to 1.10 and 1.12 for 600 °C GF and 700 °C GF, respectively. Additionally, 400 °C GF showed an I_D_/I_G_ ratio of 0.78, slightly lower than that of pristine GF, possibly due to the removal of surface impurities during heat treatment.

**Figure 4:**
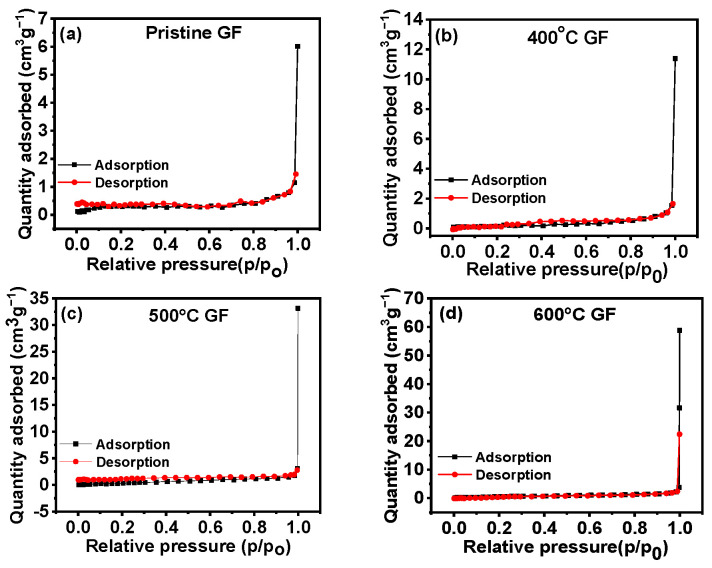
Adsorption–desorption curves for (**a**) pristine GF, (**b**) 400 °C GF, (**c**) 500 °C GF and (**d**) 600 °C GF.

Surface area values varied for GFs treated at different temperatures, and nitrogen adsorption-–desorption curves for different heated GFs are given in Figure 4. Table 1 lists the surface area values for all GFs, revealing slight increases in the active surface areas of the GFs with heat treatment. This signifies that the surface activity of the GFs will increase with heat treatment due to an increase in the active surface area and defects in heat-treated GFs.

**Figure 5:**
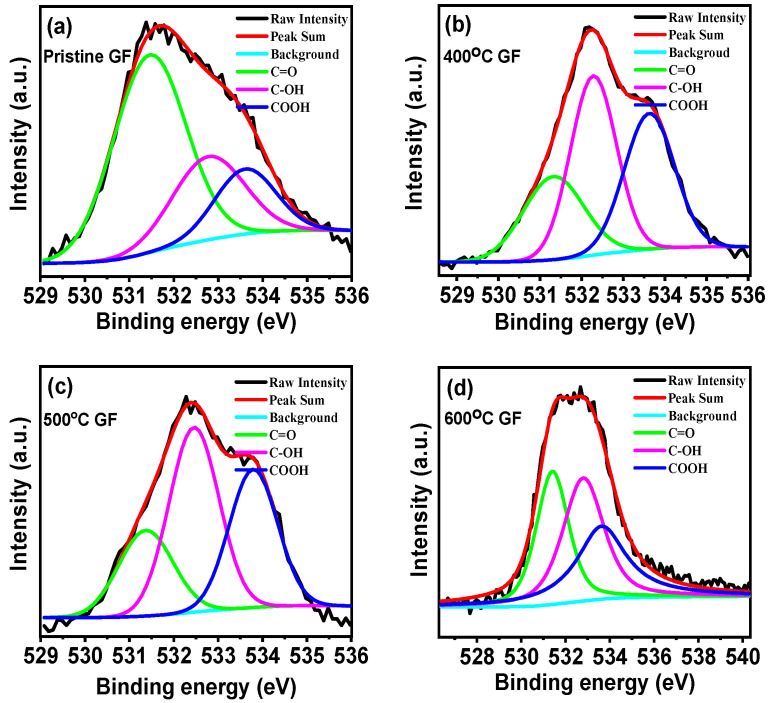
Deconvoluted O1s XPS spectra of (**a**) pristine GF, (**b**) 400 °C GF, (**c**) 500 °C GF and (**d**) 600 °C GF.

Contact angle measurements showed that contact angles of heat-treated GFs were less in comparison to pristine GF, as given in supplementary information (SI) (Figure S1). The lowest contact angle was 130° for 600 °C GF, which was 140° in the case of pristine GF. Decreases in the contact angle with heat treatment were due to decreases in the hydrophobicities of GFs’ surfaces because of decreased surface tension. Thus, heat treatment increases the wettability of GFs’ surfaces. An XPS study was used to understand the further details of surface functionality changes in GFs due to heat treatment at different temperatures. The XPS spectra of pristine GF, 400 °C GF, 500 °C GF and 600 °C GF are shown in SI (Figure S2). Deconvoluted O1s XPS spectra of pristine GF, 400 °C GF, 500 °C GF and 600 °C GF are shown in Figure 5. The deconvolution of O1s spectra was performed for C=O, C-OH, COOH and H-OH oxygen bonded groups, and the corresponding binding energies are 531.5, 532.5, 533.8 and 534.8 eV, respectively. Here, the H-OH bond has a negligible contribution to the oxygen functionalities of GF, the other three oxygen functional groups are presented in different percentages in pristine, and the heat-treated GFs are as given in Table 2. The absence of the H-OH bond was attributed to the heat treatment of GFs at temperatures more than 100 °C. The percentage of oxygen present increased with increases in the heating temperature. Also, it was observed that with heat treatment, the C=O content decreased but C-OH and COOH functionalities increased. The overall oxygen content present in GFs can be estimated with the O/C ratio, which increased with varying temperature to a higher value. Moreover, 500 °C GF and 600 °C GF show the highest O/C ratios of 0.062 and 0.064, respectively, which were considerably higher than for pristine GF (0.036). Higher oxygen content indicates the presence of a greater active site for redox reactions; thus, heat-treated GFs will have more reactivity towards the reactions [27,31].

### 3.2. Electrochemical Characterizations

**Figure 6:**
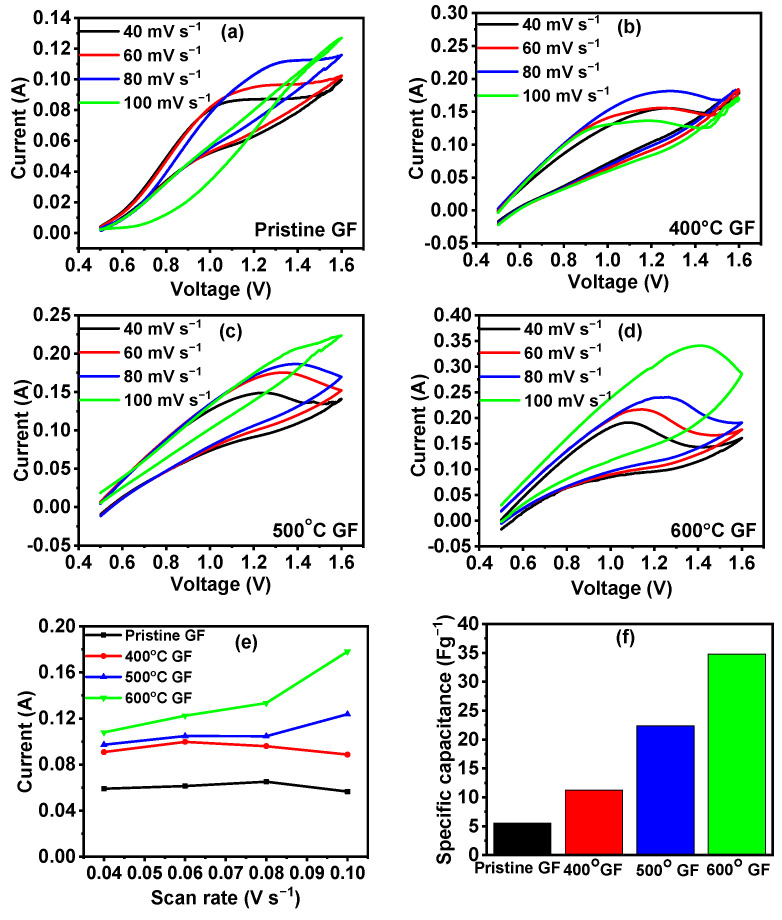
CV graphs of (**a**) pristine GF, (**b**) 400 °C GF, (**c**) 500 °C GF and (**d**) 600 °C GF in 1M FeCl_2_ and 1.5 M NH_4_Cl electrolyte; (**e**) variation in current vs. scan rate for pristine GF, 400 °C GF, 500 °C GF and 600 °C GF at 40 mV s^−1^; (**f**) specific capacitance for pristine GF, 400 °C GF, 500 °C GF and 600 °C GF at 40 mV s^−1^ .

The electrochemical performances of various heat-treated GFs were evaluated by calculating their specific capacitance from CV. Figure 6a–d shows the CV curves for pristine GF, 400 °C GF, 500 °C GF and 600 °C GF, respectively. CV curves are not reversible because metallic iron formed during the redox reaction of the Fe^2+^/Fe^3+^ pair, which cannot be easily dissolved in electrolyte. This results in irreversible redox reactions. Also, the CV curves were similar to the one reported by Minakshi et al. [32] for Li(Co_0.5_Ni_0.5_)PO_4_ compound, which suggested the effect of HER on the oxidation–reduction reaction. It can be observed from these plots that the area under the curve and current responses increased from pristine GF to 600 °C GF, which was due to increased surface activity because of the heat treatment of GFs. Increased surface area and incorporated oxygen functional groups make the heat-treated GF surface more feasible for chemical reactions compared to the pristine GF. Figure 6e shows the current responses of various GFs with varied scan rates, which concluded that currents were higher in heat-treated GFs. The further specific capacitances of heat-treated GFs were higher compared to pristine GF. The specific capacitance for each GF electrode was calculated from CV curves using formula


$$\text{Specific capacitance}\;=\frac{Area\;under\;the\;CV\;curve}{Scan\;rate\;\times \;voltage\;\times \;weight\;of\;sample}$$

The specific capacitances of pristine GF, 400 °C GF, 500 °C GF and 600 °C GF were 5.5, 11.2, 22.3 and 32.5 Fg^−1^ at 100 mV s^−1^, respectively. Specific capacitances calculated at different scan rates were reported in SI (Table S1). The specific capacitances of GFs decreased with increasing scan rates. At a higher scan rate, electrolyte ions do not have sufficient time for adsorption–desorption in the internal structure and pores of the electrode [33]. Figure 5f gives the comparison of specific capacitances for all types of GFs. 

Also, it has been reported that specific capacitance is dependent on the wettability of the electrode surface. It has been reported that if electrode has a smaller contact angle then it will have better specific capacitance. A smaller contact angle signifies good wettability, which is responsible for decreasing the electrode–electrolyte contact resistance [28,34]. Here, 600 °C GF exhibited the lowest contact angle of 130° (SI—Figure S1), which resulted in the highest specific capacitance among all GFs.

Heat-treated GFs exhibited more specific capacitances, which suggests good electrochemical behavior in comparison to pristine GF. In general, for carbon materials, specific capacitance is result of two types of charge storing mechanisms, the adsorption–desorption of ions on the active surface area and redox reactions with surface active functional groups. A high value of specific capacitance means that more energy can be stored as $E=\frac{1}{2}{CV}^{2}$ [35]. Thus, an electrode with good specific capacitance can be used in RFBs as electrode features, like high surface area and good surface reactivity, which are also concerning factors for RFBs electrodes [36]. Here, all the heat-treated GFs have more specific capacitances compared to the pristine GF, so heat-treated GFs were further examined for the use of IRFB electrodes.

### 3.3. Battery Performance

**Figure 7:**
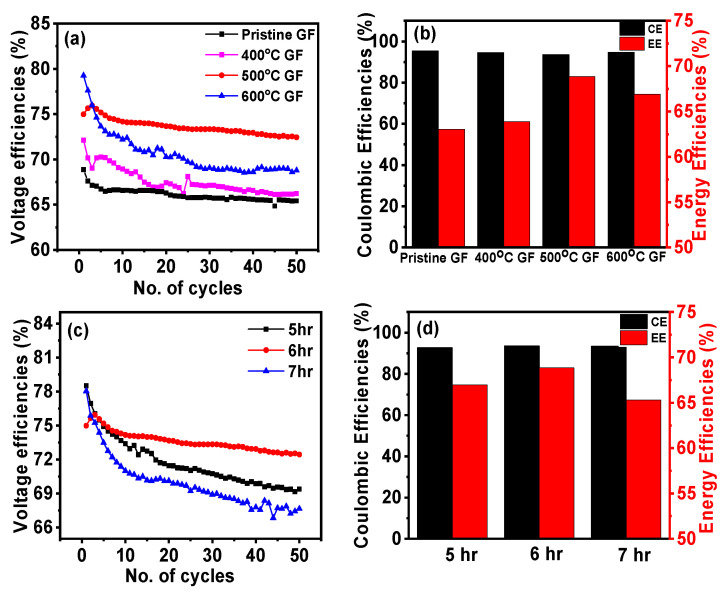
(**a**) Variation in voltage efficiency (VE) with the number of cycles for pristine GF, 400 °C GF, 500 °C GF and 600 °C GF; (**b**) Coulombic efficiencies (CEs) and energy efficiencies (EEs) for pristine GF, 400 °C GF, 500 °C GF and 600 °C GF; (**c**) effect on VE of heat-treated GF for 5, 6 and 7 h at 500 °C GF; (**d**) CEs and EEs of 500 °C GF for 5, 6 and 7 h.

Battery performance was assessed in terms of voltage efficiency (VE), Coulombic efficiency (CE) and energy efficiency (EE). A comparison of VE, CE and EE for different heat-treated GFs is shown in Figure 7a and Figure 7b, respectively. VE, CE and EE for pristine GF, 400 °C GF, 500 °C GF and 600 °C GF are reported in Table 3. Moreover, 500 °C GF consisting of a battery exhibited the highest VE, CE and EE, which were ~74%, ~94% and ~70%, respectively, for 50 charge–discharge cycles at 20 mA cm^−2^. These efficiencies were significantly higher than those of the battery consisting of pristine GF, which were VE ~66%, CE ~95% and EE ~63% at 20 mA cm^−2^. Thus, 500 °C GF showed a 7% higher EE compared to pristine GF. This performance improvement was due to an increase in the surface activity of GF due to the presence of oxygen functional groups and defects. But, surprisingly, 600 °C GF showed a 2% lower EE compared to 500 °C GF, which may be because of the negative effect of heat treatment. Surface structure was drastically affected in 600 °C heat treatment, as can be seen in the FESEM image (Figure 2d). However, this effect was not observed in electrochemical measurements, possibly due to the involvement of a small-area electrode (1 $\times \;1\;{\mathrm{cm}}^{2}$). In the case of battery evaluation, the active area of cell was 5 $\times \;5\;{\mathrm{cm}}^{2}$ which probably caused the greater involvement of a large damage structure in 600 °C GF. It can also be observed that for the very first few cycles, the VE of 600 °C GF was greater than that of 500 °C GF, but in long run, the damaged electrode structure degraded the performance. CEs did not show much variation in all kinds of electrodes and VE was defining factor of EE. A slight decrease in VE was observed with an increase in the number of cycles due to irreversible losses of electrolyte active species [37,38]. In order to analyze the effect of the heat treatment time duration, VE, CE and EE were calculated for heat-treated GFs at 500 °C for time durations of 5, 6 and 7 h. Variations in VE, CE and EE for heat-treated 500 °C GFs for 5, 6 and 7 h are shown in Figure 7c,d. It can be concluded from the plots that a 6-h heating time was the most suitable for the battery compared to 5 and 7 h, which was assumed to be because of insufficient oxygen functionalities and structural damage in the 5- and 7-h heating times, respectively. Thus, an optimized heat treatment can significantly affect the reaction kinetics of the GF and ultimately improve the IRFB’s performance. The reason behind this performance enhancement was the promoted electrochemical behavior of heat-treated GFs because of the presence of oxygen functional groups, which improved the active surface area and enhanced surface wettability. These findings were consistent with the electrochemical characterizations. 

**Figure 8:**
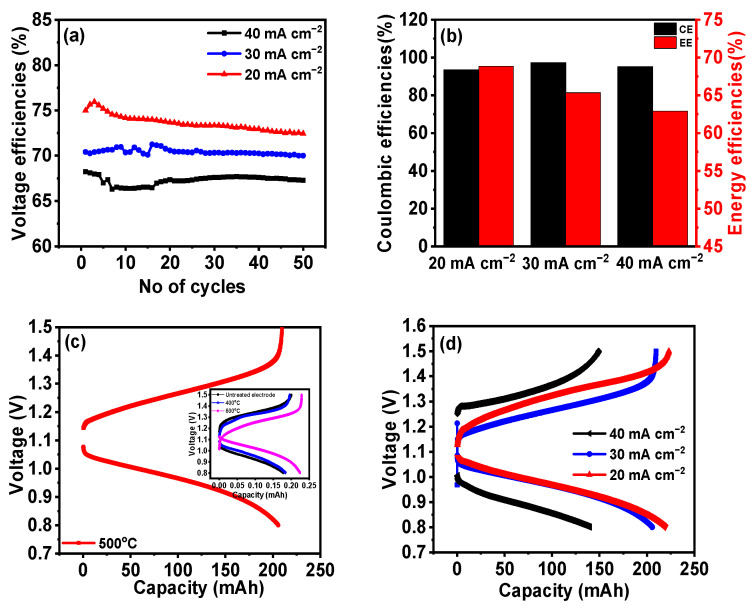
Variations in (**a**) VE, (**b**) CE and EE for 20, 30 and 40 mA cm^−2^ for 500 °C GF; (**c**) voltage vs. capacity comparison of the first charge–discharge cycles for pristine GF, 400 °C GF, 500 °C GF and 600 °C GF; (**d**) voltage vs. capacity comparison of the first charge–discharge cycle for 500 °C GF operating with 20, 30 and 40 mA cm^−2^.

**Figure 9:**
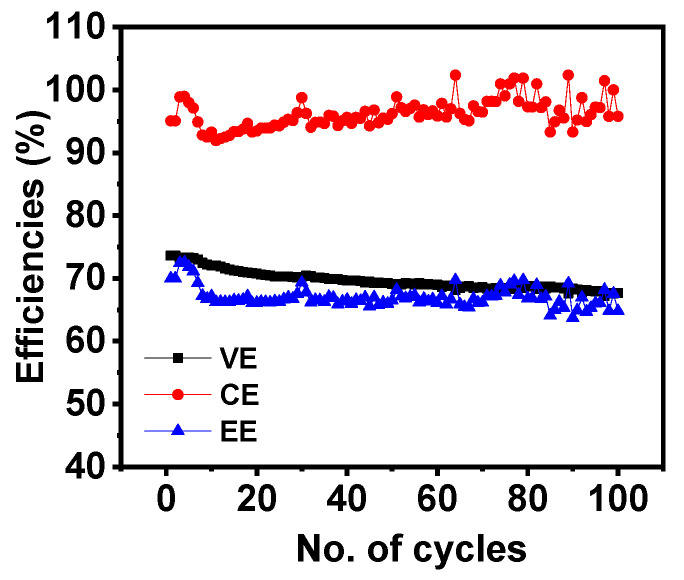
Average VE, CE and EE for 100 charge–discharge cycles of IRFBs at 20 mA cm^−2^.

Figure 8a,b gives the IRFB performances at different current densities for 500 °C GF. Increased current densities result in a decreased VE, which was due to an increase in the resistance losses at higher currents [39]. VEs, CEs, and EEs for a battery consisting of 500 °C GF electrodes, working at different current densities, are listed in Table 4. Figure 8c gives the voltage vs. capacity variation in the first cycles of pristine GF, 400 °C GF, 500 °C GF and 600 °C GF at 20 mA cm^−2^. Additionally, the 500 °C GF electrode battery showed the longest charge–discharge cycle compared to the other three kinds of electrodes, which was due to its most suitable surface chemistry. The charge–discharge cycle capacities were significantly affected due to the different surface chemistries of various heat-treated GFs, as for pristine GF, 400 °C GF and 600 °C GF, capacities were very low. In addition, an increase in current density decreased the charge–discharge cycle duration, as can be seen from the comparison of first cycle of the 500 °C GF battery at 20, 30 and 40 mA cm^−2^ (Figure 8d). This decrease was due to an increase in Ohmic losses at higher current densities. But still, the battery showed very little difference in capacity at 20 and 30 mA cm^−2^ current densities. Battery was also capable of working for 100 charge–discharge cycles with no significant performance degradation. Battery performance for 100 cycles is shown in Figure 9, and the average VE, CE and EE for 100 cycles were ~69%, ~96% and ~67%, respectively. 

## 4. Conclusions

This study involves the heat treatment of GF electrodes and analyzes its effect on the performances of IRFBs. GFs were furnace heat-treated at 400, 500, 600 and 700 °C for 6 h with a ramp rate of 10 degrees per minute. TGA and FESEM results suggested that the 700 °C GF was drastically degraded due to high-temperature heating, making it mechanically unstable for battery use. FESEM images of the 600 °C GF also showed some carbon degradation due to oxidation at high temperatures. XPS, Raman spectroscopy, and surface area results concluded that heat treatment significantly improved the surface activity of the 400 °C GF, 500 °C GF and 600 °C GF compared to pristine GF. Specific capacitance calculated from cyclic voltammetry was highest at 34.8 F g^−1^, compared to only 5.5 Fg^−1^ for pristine GF at 100 mV s^−1^. This performance improvement was due to the insertion of oxygen functionalities, and the XPS suggested that the highest O/C ratio was 0.064 in the 600 °C GF, compared to a ratio of 0.032 for a pristine GF. Although the specific capacitance was higher in the 600 °C GF, the battery performance was most improved for the 500 °C GF. This was possibly because the structural degradation of the 600 °C GF reduced the battery performance, which was not present in the case of the 500 °C GF. Also, the O/C ratio of 500 °C GF was 0.062, which was comparable to 600 °C GF, so the 500 °C GF electrodes were more suitable for longer battery performance. The battery consisting of the 500 °C GF electrodes showed ~74% VE, ~94% CE and ~70% EE for 50 charge–discharge cycles at 20 mA cm^−2^. The battery can also operate at high current densities of 30 mA cm^−2^ and 40 mA cm^−2^. The average VE, CE, and EE for 100 charge–discharge cycles of IRFB were ~69%, ~96% and ~67%, respectively.

## Figures and Tables

**Table 1:** N_2_ absorption–desorption surface area and Raman spectra I_D_/I_G_ ratio for all the GFs.

Sample Name	Pristine GF	400 °C GF	500 °C GF	600 °C GF
Surface Area (m^2^ g^−1^)	0.063	0.081	0.192	0.254
I_D_/I_G_ ratio from Raman spectra	0.89	0.78	1.10	1.12

**Table 2:** Calculated percentages of different oxygen functional groups present in pristine and heat- treated GFs from XPS deconvoluted peaks.

Sample	C1s (%)	O1s (%)	O/C Ratio	C=O (%) (531.5 eV)	C-OH (%) (532.5 eV)	COOH (%) (533.8 eV)
Pristine GF	96.53	3.47	0.036	59.11	14.42	16.29
400 °C GF	95.36	4.64	0.049	30.87	37.30	31.82
500 °C GF	94.16	5.84	0.062	25.04	41.04	33.91
600 °C GF	94.03	5.97	0.064	22.66	44.88	32.43

**Table 3:** Average values of voltage efficiency (VE), Coulombic efficiency (CE) and energy efficiency (EE) for pristine GF, 400 °C GF, 500 °C GF and 600 °C GF.

Sample Name	Pristine GF	400 °C GF	500 °C GF	600 °C GF
Voltage efficiencies (VE) (%)	66.09	67.55	73.61	70.58
Coulombic efficiencies (CE) (%)	95.36	94.56	93.55	94.73
Energy efficiencies (EE) (%)	63.03	63.89	69.50	66.89

**Table 4:** Summary of the VE, CE and EE values for different current densities for IRFB consisting of 500 °C GF.

Current Densities	VE (%)	CE (%)	EE (%)
20 mA cm^−2^	73.62	94.55	69.60
30 mA cm^−2^	67.23	97.29	65.35
40 mA cm^−2^	66.09	95.20	63.00

## Data Availability

The data presented in this study are available on request from the corresponding author.

## Supplementary Materials

The following supporting information can be downloaded via this link: https://www.mdpi.com/article/10.3390/nano14090800/s1, Figure S1: Contact angle measurements for (a) pristine GF, (b) 400 °C GF, (c) 500 °C GF and (d) 600 °C GF; Figure S2: XPS spectra of pristine GF, 400 °C GF, 500 °C GF and 600 °C GF; Table S1: Calculated specific capacitances for pristine GF, 400 °C GF, 500 °C GF and 600 °C GF at different cyclic voltammetry scan rates.
