# Supplementary material for: Physiochemical and Electrochemical Properties of a Heat-Treated Electrode for All-Iron Redox Flow Batteries
Source: Nanomaterials (Basel). 2024 May 5;14(9):800. doi: 10.3390/nano14090800 (PMC11085079; doi:10.3390/nano14090800)
Supplement: Supplementary file 1 [file nanomaterials-14-00800-s001.zip › nanomaterials-2974350-supplementary.pdf]

Physiochemical and Electrochemical Properties of a Heat-Treated Electrode for All-Iron Redox Flow Batteries

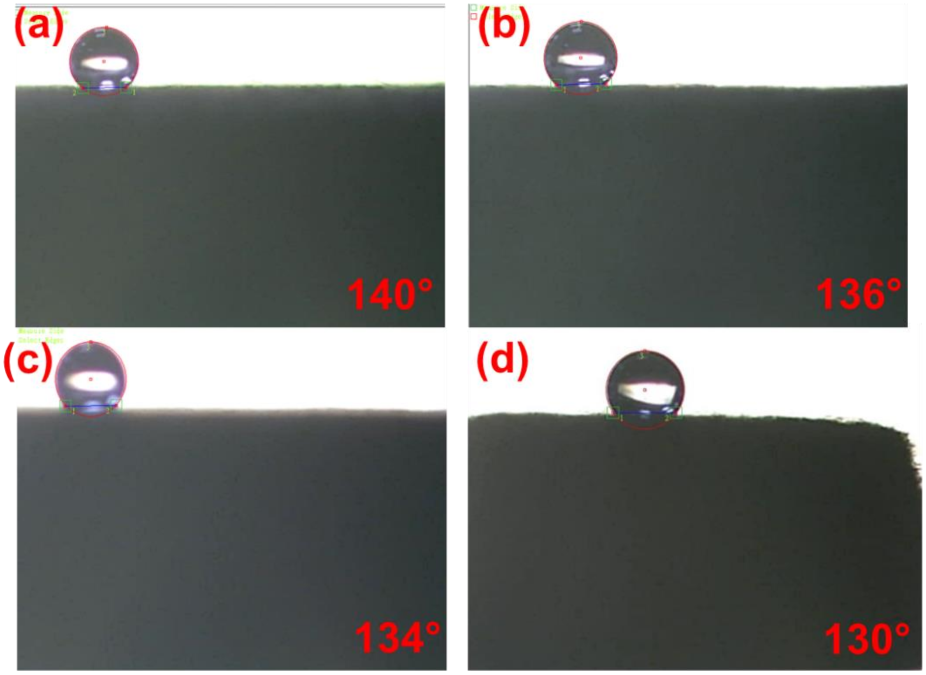

Figure S1. Contact angle measurements for (a) Pristine GF; (b) 400 °C GF; (c) 500 °C GF; (d) 600 °C GF.

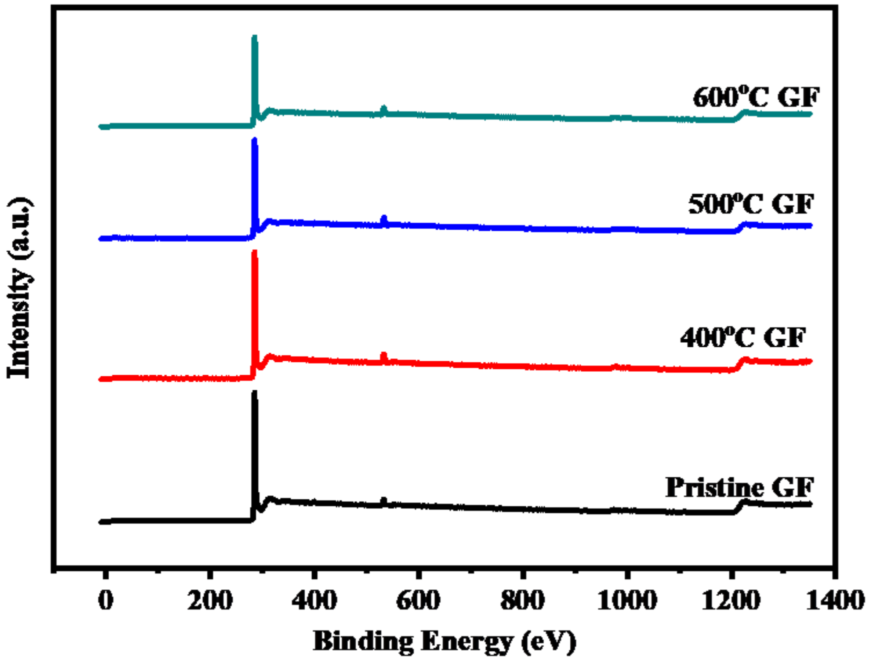

Figure S2. XPS spectra of pristine GF, 400 °C GF, 500 °C GF, 600 °C GF.

Table S1. Calculated specific capacitances for pristine GF, 400 °C GF, 500 °C GF, 600 °C GF at different cyclic voltammetry scan rates.

**Scan rate** / **Sample name**	**40 mV s^{-1}**	**60 mV s^{-1}**	**80 mV s^{-1}**	**100 mV s^{-1}**
Pristine GF	19.5 Fg^{-1}	15.1 Fg^{-1}	11.8 Fg^{-1}	5.5 Fg^{-1}
400°C GF	31.5 Fg^{-1}	26.5 Fg^{-1}	16.5 Fg^{-1}	11.2 Fg^{-1}
500°C GF	40.5 Fg^{-1}	30.3 Fg^{-1}	23.9 Fg^{-1}	22.3 Fg^{-1}
600°C GF	52.4 Fg^{-1}	39.4 Fg^{-1}	34.8 Fg^{-1}	32.5 Fg^{-1}
